# Parenting Intervention for Psychological Flexibility and Emotion Regulation: Clinical Protocol and an Evidence-Based Case Study

**DOI:** 10.3390/ijerph19095014

**Published:** 2022-04-20

**Authors:** Juan M. Flujas-Contreras, Azucena García-Palacios, Inmaculada Gómez

**Affiliations:** 1 Department of Psychology, University of Almeria, 04120 Almeria, Spain; igomez@ual.es; 2 Health Research Centre (CEINSA/UAL), University of Almeria, 04120 Almeria, Spain; 3 Department of Psychology, University Jaume I, 12071 Castellon, Spain; azucena@uji.es; 4 CIBER of Physiopathology of Obesity and Nutrition (CIBERobn), Instituto de Salud Carlos III, 28029 Madrid, Spain

**Keywords:** adolescent, family, oppositional defiant disorder, third-wave therapy

## Abstract

Psychological flexibility has been found as a protective factor for several psychological problems, including the field of parenting. The present study aims to illustrate a clinical protocol, session by session, for the promotion of parental psychological flexibility and emotion regulation in a case study. The clinical protocol is based on third-wave behavior therapy in a brief intervention of four sessions. The intervention is presented in a clinical case of a mother with a child diagnosed with Oppositional Defiant Disorder. Both mother and child experienced problems with emotional regulation and psychological flexibility. The results show clinically significant improvements in psychological flexibility, emotional regulation, and stress parenting in the mother both after the intervention and at follow-up. In the child, emotional perspective-taking skills, acceptance, and valued actions improved. The case illustrates in detail the application of different strategies of acceptance, mindfulness, emotion regulation, and emotional defusion applicable to parenting. Clinical implications are discussed.

## 1. Introduction

Psychological flexibility is defined as the disposition to remain in contact with unpleasant experiences and private events, fully and consciously, in the direction of values [1]. This skill has been explored as a positive factor in mental health, anxiety, or depression. Specifically, psychological flexibility allows individuals to adapt for greater personal functioning, both cognitively and behaviorally. This makes psychological flexibility a protective factor with respect to mental health [2,3]. Psychological flexibility is a response and a coping style for distressing situations in life. In contrast to other perspectives in which cognitive or behavioral avoidance is a valid strategy, psychological flexibility aims to enhance functional patterns with acceptance-based coping strategies. Previous studies have shown that emotional suppression and avoidance coping strategies have paradoxical effects [4,5]. It is therefore not surprising that psychological flexibility is involved in parenting and bringing up children [6], and this is called parental psychological flexibility. In fact, several studies relate psychological flexibility as a protective factor with respect to the stress that parenting may entail. It is also a mediating factor in parenting styles in parent–child interactions [7]. A flexible pattern in parenting has been shown to be related to fewer externalized and internalized problems, better parenting practices, personal and family adjustment, and reactivity [8,9,10,11]. Parenting focused on the present moment is related to better adaptive emotion regulation and attachment in children [12], as well as more emotion regulation, compassion, and psychological flexibility in adolescents [13]. Parenting styles in turn affect the development of psychological flexibility in their children, and authoritarian styles are related with less psychological flexibility in children [14]. Regardless of psychological flexibility, parenting practices have an influence on the psychological well-being of children [15]. Specifically, it has been found that authoritarian or permissive styles are directly related to aggressive behaviors in children [16,17]. Similarly, authoritarian and neglectful parenting styles are related to antisocial behaviors [18] and irritability or defiant disorders [19]. This relationship is also found with the academic performance of the children [20,21].

Acceptance and Commitment Therapy (ACT; [1]) aims to enhance psychological flexibility. In this sense, the training in this skill in ACT is supported by six interconnected skills: acceptance, defusion, mindfulness, self as context, actions, and values. These skills can be understood as three flexible response styles: a response style that is willing to be in contact with their uncomfortable feelings (open), focused on the present moment (aware), and engaged in their actions in the direction of values (active) [1]. ACT is part of a group of therapies called third-wave behavior therapies that emphasize the contextual and functional value of behavior [22]. The strategies of this therapy are based on the behavioral principles of learning and language analysis through the Relational Frame Theory (RFT; [23]).

Psychological flexibility, when included in parenting, is a way to relate with private events (emotions, thoughts, feelings, etc.). It can therefore be understood as an adaptive strategy for regulating emotions focused on acceptance for a value-driven purpose. ACT has not been developed as a parenting intervention as such, but it has made use of strategies and components to intervene with parents [24]. A review by Byrne et al. [25] noted that the application of ACT in parents has shown positive effects in children with ASD, chronic pain, in medical problems (such as acquired brain injury, cerebral palsy, asthma, diabetes, deafness, etc.), and anxiety. In addition, mindfulness training programs for mothers have shown reduction in aggressive behaviors in their children with autism spectrum disorders (ASD) [26]. From an ACT perspective, Blackledge and Hayes [27] suggested that emotions per se do not cause maladaptive emotion regulation, but are the consequence of attempts at regulating these emotions and interference of these actions in one’s life, within the social, cultural, and verbal context. In this regard, the concept of flexible emotion regulation emphasizes the use of emotion regulation strategies adapted to contextual demands, and considers the coherence of the strategy with the direction of personal goals [28]. A study by Seligowski and Orcutt [29] found that in Gross’s [30] model, emotional distancing strategies are a factor in proneness to emotion.

Emotion regulation of parents in terms of frequency, duration, and valence affects the emotional development of their children and family dynamics [31]. Emotion regulation by parents with children with or without a psychological disorder also differ [32]. It should be considered that emotion regulation styles are not only learned by children following parental models, but that those of children also affect their parents [33,34]. Similarly, parenting styles affect the emotional development of children and family interaction [35,36]. Thus, the response of parents to the emotional reactions of their children is important to their development. Parental response to their children’s behavior based on emotional acceptance and validation positively affects emotion regulation and reactivity [37], whereas responses such as invalidation can favor the development of psychological problems in their children [38].

That is the reason why the intervention in families must consider the parents’ abilities, not only of parenting, but also their abilities to face their emotions, thoughts, or sensations in situations of managing their children’s behavior or emotions. The aim of this study is to illustrate the application of a clinical protocol for the promotion of parental psychological flexibility and emotional regulation through third-wave behavior therapy (or contextual therapy) strategies in parents of children with psychological diagnosis in a secondary analysis. This clinical protocol has been shown to be effective in improving psychological flexibility, emotional regulation, and parental stress in different formats [39,40,41]. Specifically, this study addresses this clinical protocol in the case of a mother with a child with Oppositional Defiant Disorder [24], as well as explores the effects of the intervention on children. Considering evidence-based treatments for adolescents with behavioral disorders, level one treatments (working well) are those that combine behavioral therapy, cognitive–behavioral therapy, and family therapy [42]. Likewise, Division 53 of the APA [43] indicates as experimental phase treatments some intervention programs with third-wave therapies, such as mindfulness or Dialectical Behavioral Therapy. The role of parenting skills, the parent–child relationship, social support, and parental emotional regulation are shown to be potential factors (risk/protective factor) that may influence the development of Oppositional Defiant Disorder [44,45,46,47]. Therefore, it is relevant to provide parental interventions in emotional issues such as acceptance or emotional distancing that promote functional and constructive parenting skills. This paper illustrates the application of an intervention, mainly based on ACT strategies, due to the difficulties related to the mother’s rumination and cognitive fusion, which are hypothesized to be the basis of the family interaction problems. It is expected that the effects of the intervention with the mother will also improve the child’s behavior, even if no intervention was made directly with the child, as observed in previous studies [48].

## 2. Methods

### 2.1. Patient Presentation

David’s parents sought help worried about the problems they were experiencing with their son. Over the prior year, David’s coexistence and behavior problems were becoming worse, and his parents were looking for a solution so that David “*learns to control his emotions*”. The parents felt they had tried many parenting strategies and had no more resources to deal with their child’s behavior problems. 

David is a 12-year-old Spanish boy with Oppositional Defiant Disorder [49], diagnosed at age 8. This disorder is characterized by a pattern of anger, defiance, or vengefulness that affects the person individually or their social environment (family, school, etc.), persisting for at least six months. In the year before David’s parents decided to seek help, David’s behavioral problems worsened. His symptoms were mainly explosive and aggressive behavior associated with frustration tolerance, especially at home (e.g., losing in video games or not being able to leave home). Those behaviors led his parents to use strategies of control of stimulus and punishment, but those strategies were not efficient. His parents felt discomfort associated with their family problem that made coexistence and family dynamics increasingly complex. All participants gave their informed consent before starting the study and they did not receive any financial incentive for study participation. This study received the ethical approval of the Andalusian Health Service’s Almeria Research Committee (reference: PI-REFLEX-ESFA-19, approved: 24 June 2020).

### 2.2. Clinical Case History

David lives with his mother (45 years old), his father (45 years old), and his older sister (13 years old) in a single-family house in a municipality near to the provincial capital. They are a middle-class family. The mother is an independent worker as a support teacher and has higher education. She has been diagnosed with fibromyalgia and asthma. The father has basic education and works as a freelancer with a music studio at home. Both consider that they have received little support to face the problem of their son.

David was officially diagnosed with Oppositional Defiant Disorder [24] at age 8, without having history or comorbidities of other disorders. Aripiprazol was prescribed from the beginning, which with psychological support can stabilize behavioral problems. However, these problems reappeared at age 11, mainly in the family context with defiant attitude, irritability, impulsiveness, and vengeful behavior with their relatives. 

The pregnancy proceeded normally, and he was born with branchial paralysis. There is no history of medical illness. In the family there is a history of impulse control problems on the maternal side (the father of the mother), with strong attacks of anger and aggressiveness, aggravated by alcohol abuse.

David has always been a contentious boy, with low academic motivation. He has some basic housework responsibilities, although his mother ends up fulfilling his responsibilities. At times he has been saturated with activities that he did not choose himself (e.g., playing the piano). The parents describe a family environment in which each is doing their own things and sharing little family time. The parents tried to control David’s behavior by repeating the rules and even insulting him, yelling at him, and punishing him by withdrawing privileges.

Before starting the intervention, the parents signed the informed consent. All the data presented in this case study are masked to safeguard confidentiality.

### 2.3. Assessment

#### 2.3.1. Parent Outcome and Family Functioning 

The information reported in the clinical history section was obtained in an initial interview attended by both parents. After that, the following instruments were applied. 

Parental Acceptance Questionnaire (6-PAQ) [50] was applied to assess parental psychological flexibility and three related behavioral styles: open, aware, and active. The Spanish version of the scale consists of 16 items on a four-point Likert scale. The overall scale scores are between 4 and 64. The Spanish version of the instrument has a Cronbach’s alpha of 0.81 [51] (RCI total score: 6.83). (To estimate the clinical efficacy of the intervention, the Reliable Change Index (RCI) was calculated using the method of Jacobson and Truax [52] for all variables (see baseline results section for more details.) 

Acceptance and Action Questionnaire-II (AAQ-II) [53,54] measures experiential avoidance in 7 items on a 7-point Likert scale. The overall scale scores are between 7 and 49. The Spanish version of the instrument has a Cronbach’s alpha of 0.88 (RCI total score: 10.63).

Difficulties in Emotion Regulation Scale (DERS) [55,56] was used to evaluate treatment effects on emotion regulation. This scale measures a total score and 5 emotion regulation processes in 28 items on a 5-point Likert scale. The overall scale scores are between 5 and 140. The total score for the Spanish version of the instrument has a Cronbach’s alpha of 0.91 (RCI total score: 12.91).

Parenting Stress Scale (PSS) [57,58] was used to asses stress related to parenting. This scale consists of two dimensions in 12 items rated on a 5-point Likert-type scale: baby rewards and parent stressors. The overall scale scores are between 5 and 60. The Spanish version of the instrument has a Cronbach’s alpha of 0.77 (RCI total score: 7.98).

Satisfaction with Life Scale (SWLS) [59,60] was used to assess general satisfaction with life. It consists of 5 items on a 5-point Likert scale. The overall scale scores are between 5 and 25. The Spanish version of the instrument has a Cronbach’s alpha of 0.88 (RCI total score: 5.7).

Finally, the Parenting Style Questionnaire (CEEP) [61] was used to assess parenting difficulties at pre-test. This scale consists of five dimensions in 110 items: family dynamics, emotional competences, parent role, parenting style, and parenting practices. The Spanish version of the instrument has a Cronbach’s alpha of 0.92. 

#### 2.3.2. Parent Process Outcome

To evaluate the progress of the intervention, at the beginning and at the end of each session mood, coping and consistency in valued actions were assessed. Mood was measured by asking “How do you feel right now?” followed by a 5-face visual scale that has been used in previous studies [62]. Coping perception was assessed using the question “How able do you feel to address your concerns about your children right now?”. At pre-session, the implication in valued direction action was assessed on a 10-point Likert scale (we transformed this score to a 5-point scale to make it comparable to the other process outcomes). These questions were developed ad hoc for a momentary assessment of the person’s progress in the intervention.

#### 2.3.3. Child Outcomes

Strengths and Difficulties Questionnaire (SDQ) [63,64] was used to assess treatment effects in David. This scale consists of a total score and 5 factors: emotion symptoms, behavioral problems, hyperactivity, problems with peers, and prosocial behavior. The overall scale scores are between 0 and 50. The Spanish version of the instrument has a Cronbach’s alpha of 0.77 (RCI total score: 8.77).

David’s psychological flexibility was assessed using the Avoidance and Fusion Questionnaire for Youth [65,66] that measures avoidance and cognitive fusion; it has a Cronbach’s alpha of 0.87. The overall scale scores are between 0 and 68 (RCI total score: 0.76).

The Willingness and Action Measure [67,68] was used to assess acceptance and valued-oriented actions. The overall scale scores are between 5 and 70. The Spanish version of the instrument has a Cronbach’s alpha of 0.78 (RCI total score: 11.65).

#### 2.3.4. Measure of Intervention Satisfaction

Client Satisfaction Questionnaire [69,70] was used to assess general satisfaction with the treatment. It consists of 8 items on a 4-point Likert scale. 

Acceptability, usability, and interest of the clinical protocol were evaluated with 3 questions on a 4-point Likert scale, developed ex post facto. 

### 2.4. Baseline Assessment Results

The Jacobson and Truax [52] method was used to assess the clinical changes of the intervention. Specifically, the Reliable Change Index (RCI) was calculated following the “c” criteria. To obtain the cut-off score, the mean and standard deviation of the normative sample (of the instrument validation population) and of the mother were used. A score was considered “recovered” if its score on the post-test changed with respect to the RCI value and if it was lower than the cut-off score. It was classified as “improved” if it was a change in the RCI value but not in the cut-off score.

At pre-test, we found that the mother has difficulties to maintaining a parenting style with psychological flexibility, especially in her abilities to behave in an open and aware response style. That is, high scores were obtained on the 6-PAQ scale. For the AAQ-II score we observed high experiential avoidance. We found high scores in difficulties to regulate her emotions, especially for using acceptance, interference with goal-oriented behaviors, and limited access to emotion regulation strategies. Furthermore, a high score was found in parenting stress, both stressors and rewards. Satisfaction with life level was low compared to the normative mean score of the instrument. 

Regarding the mother’s parenting style, difficulties focused mainly on an excessive parental role and emotional competences. That is, high scores were obtained in these competences of parenting in relation to the mean score of the instrument. At a qualitative level, these difficulties were found as excessive burden, impulsivity, lack of perception, and management of emotions. On the other hand, we found problematic scores in terms of how she interacts with her child to manage disruptive behaviors and uses adaptive corrective strategies in her son. As for family dynamics, the presence of conflicts in family environment stands out. The predominant educational style in the mother is permissive.

The father’s pre-test assessment showed high scores of parental psychological inflexibility. In addition, scores above the normative mean in emotional regulation difficulties (DERS) were found, especially in his attention to emotions and access to strategies for emotion regulation. Like the mother, above-average scores on parental stress were found.

Finally, concerning David’s problem assessment through the SDQ, we found that the mother reported higher scores (clinical scores) of emotional symptoms than the father, while the father reported higher levels (clinical scores) of behavioral problems. Both parents reported hyperactivity and problems with peers at a normal level. Additionally, David’s self-report questionnaires showed high scores of cognitive fusion, experiential avoidance, and action difficulties in distress situations. 

### 2.5. Case Conceptualization

Due to of all the above, we considered that it was necessary to carry out an intervention with David’s parents to improve their parenting skills and emotion regulation strategies to face distress situations with their son. David’s last episodes of impulsive and aggressive behaviors were generating a coercive system in family dynamics. Based on the parents’ difficulties, a parenting intervention was proposed for both, although for work reasons the father only went to the first session. 

On the one hand, the mother was acting in a rigid and fused way, following her beliefs and thoughts of: “my son is ill and cannot control himself”, “all my son’s actions are revengeful”, “my son’s problem is an inheritance from my father”, or “he doesn’t care about everything”. All these private events were maintaining her coherence to explain the beginning and maintenance of the problem, locating the locus of control externally. When David’s behavior problems happened, the parents impulsively described all these thoughts, leading to some emotional invalidation. On the other hand, David showed ruminative responses in situations of frustration. The most frequent thoughts were “everything sucks!” and “what a disgusting life!”.

Given the literature reviewed of the effects of parents’ emotion regulation and psychological flexibility skills in their children, we hypothesized that the proposed intervention would foster parenting skills and, similarly, improve David’s emotional well-being. 

### 2.6. Treatment

The intervention took place in a public healthcare context, specifically in the infant’s and children’s mental health unit in the province of Almeria (Andalusia, Spain). Group treatment was administered in four two-hour weekly sessions (total of eight hours over one month) and was attended by four more mothers. Although the intervention was carried out as a group, the initial interview and follow-up were conducted individually.

The goal of the clinical protocol was developed following third-wave strategies to promote parental psychological flexibility and emotion regulation for coping with situations or contexts related to bringing up children who can generate stress. Specifically, this clinical protocol makes use mainly of ACT [1] strategies. The clinical protocol was developed by the authors of this article. The intervention was applied by a psychology Ph.D. student with training and experience in the application of these therapies in children, adolescents, and families. The protocol was supervised by two psychology department heads and controlled with a checklist to ensure all the exercises were performed. The clinical protocol was framed within a metaphor written for the purpose called “the Parenting Forest”, based on which a series of exercises were performed for improving parental emotion regulation strategies with acceptance, focusing on the present and directed at actions in the direction of their values. Table 1 shows the clinical protocol contents and exercises.

#### 2.6.1. Session 1

The treatment program was introduced with a presentation and ice-breaker exercise memorizing the names and some characteristics of the members of the group. In general, the participants did not remember the characteristics of the others and we made use of this to make an analogy with the concept of mindfulness. Then, the “Forest road” metaphor was told. In this metaphor, they were asked to imagine that they were walking through a forest and at the end of the road they would find “what was most important in their lives”. While they were walking, the forest became dark, and things appeared that upset them. Then, we asked the mothers whether they should go back to the beginning of the forest or keep on to the end of the road. Later, we discussed what everyday things they do with their children to get them closer to or farther away from the end of the road. In this exercise, it was intended to identify what emotions, thoughts, feelings, or stimuli cause them to become upset. Thus, the parents were able to identify the main sources of distress in bringing up their children and what they were doing to palliate it, by working from creative hopelessness. Attempts at avoidance maintained by negative reinforcement have paradoxical effects in raising children. If it does not appear, it is important to introduce the concept of acceptance in this metaphor and differentiate it from resignation. 

Then, some pebbles were distributed to the participants and they were told that they represented thoughts and emotions that appear in complicated situations or that cause them distress, and then they performed the “The mind is a lake” exercise (inspired by “The mind is a jar” exercise by [71]). In this exercise, the parents were asked to put the pebbles in a jar of water and name emotions and thoughts as they did so. Then they shook up all the stones causing a whirlpool, and they could see that this way they could not distinguish or see thoughts and/or feelings clearly, and it is harder to regulate behavior. This exercise emphasizes that the thoughts and emotions form part of the mind, that they are there and are part of our nature. Strategies directed at eliminating or diminishing these private events may be counterproductive. 

In continuation, the strategies the parents used to regulate their emotions were explored, and the “mindfulness to breathing” exercise was performed as a useful strategy for identifying emotions and paying attention to them.

Finally, values were clarified and general goals posed based on those values using “The garden” metaphor [72]. Valued aspects are presented in this metaphor (children and two chosen by the mother herself) as plants that must be cared for by a gardener (the mother). Thus, goals were differentiated from the values as the basis for concrete actions to achieve the goals proposed. The work on committed actions and values is ideographic and guides the treatment. 

For homework, they were asked to practice full attention with their children for at least 30 min, avoiding distractions and thinking about what specific actions would meet their goals toward values.

#### 2.6.2. Session 2

The purpose of the second session was to provide the families with emotion regulation skills related to acceptance, mindfulness, and decision-making (problem-solving). 

It began by reviewing “the garden” metaphor in which the difficulties that could come up while practicing full attention were discussed, and a series of specific actions for achieving the goals were set (among them trying to include practicing full attention in the time they devoted to their children). 

Then, the “Body scan” mindfulness exercise was practiced. This exercise was intended to expand the parents’ mindfulness skills by helping them to identify their physical sensations related to emotions. It was explained that these physical sensations can help identify the emotions that drive behavior and can be a “warning signal” to make space. 

Based on the barriers found in completing the homework assigned in the previous session, the difficulties in regulating emotions and balancing actions carried out were discussed. Then, the “wise mind” (Linehan, 1993) exercise was presented. In this exercise, mental states or mindsets were distinguished: the rational mind, which is guided by logical thought, and the emotional mind, where mood and feelings at the moment guide behavior. At this point, some example behaviors were suggested which use each of these two minds, and their usefulness was discussed, always validating both mindsets depending on the context. If they did not arrive at the conclusion of an intermediate approach on their own, we presented the “wise mind” as a state in which the emotional and rational mind are balanced, and personal values take the lead in guiding actions. From that moment on we reminded them to use the wise mind to promote actions based on values with acceptance (see Appendix A for a clinical dialogue with the mother).

Finally, a defusion exercise which we call “The star observatory” was practiced. Its purpose is to strengthen perspective-taking in private events. The mother was asked to imagine a thought or an emotion that generated distress in parenting. Then, she should imagine this emotion in a starry night sky, like a constellation on which she should concentrate all her attention. She was asked to imagine different physical characteristics of this constellation (shape, color, luminosity, etc.) and locate it from different perspectives. To finish, she was supposed to distance herself from this constellation while observing that it was part of a whole heaven of constellations. This was intended to show the participant how to distance herself from that thought, reframing this private event in a hierarchy with respect to herself as just another experience forming part of her whole context. It is expected for this type of exercise to help de-literalize private events, making it easier for the parents to take action in the direction of their values, and not necessarily toward what they think or feel: that is, make space for acceptance. This exercise connects with the “Forest road” metaphor and the “Mind is a lake” exercise. 

As homework, they were asked to continue practicing informal full attention in some activity with their children and for themselves. A list of informal full attention activities was proposed (eating, showering, observing sounds, etc.). Finally, the garden metaphor was taken up again, telling them that they needed to keep working on the garden.

#### 2.6.3. Session 3

In the third session we reviewed the skills practiced and how they could be applied in a functional analysis of the children’s and the parents’ own behavior. Afterwards, homework was reviewed, and problems found were evaluated. 

The session started with an exercise on mindfulness to sounds. The exercise began with meditation concentrating on the present moment. The mothers were asked to concentrate on the sounds while a track was played with sounds from nature. As in the rest of the mindfulness exercises, they were reminded of the importance of paying full attention, without judging the experience.

Later, actions were reviewed again with the “Garden” exercise. This way, we evaluated progress during the sessions and identified the barriers that came up when these actions were carried out. Based on the problems found during the week, they were asked to identify a difficult situation with their child and make a functional analysis with the aid of a sheet. 

First, they were asked to describe the situation but not judge it. The difference between describing and judging an experience should be emphasized. A functional analysis was conducted of both parents’ and children’s behavior, keeping in mind background factors, problem behavior in terms of actions, private events, and physiological reactions, and their consequences in these three terms. At this point, they reflected on whether the reaction was consequent with their values, or if on the contrary, it was an impulsive response and they had been fused with private events when they acted. They were asked how the skills learned during the program could be employed in each of the links in this chain of behavior. As an antecedent control, the mindfulness exercises may be of help in anchoring in the present, observing the experience without judging it, and becoming aware of the private events that are related to the reactions. The defusion and acceptance exercises can be of help in taking a perspective on thoughts and emotions at the moment, so reactions are not driven by private events, but with a space for their acceptance. Finally, the garden exercise regards values and actions which are the guide for identifying whether reactions and consequences are in line with personal values and the sense they make in their own lives. The goal of facilitating functional behavior analysis associated with the skills worked on in the program is combined with parental emotion regulation by building up a flexible response pattern in parenting. 

Another defusion exercise called “The cascade of emotions” was carried out. As in the previous session, the purpose was to de-literalize private events. The participants were asked to imagine different thoughts that arise in an upsetting situation and to write them down or draw on leaves that run along the course of a river. These exercises were also intended as mechanisms for showing the mother how to lessen her reactivity to private events so she would be able to relive them without needing to judge or react to them.

The session ended with the “Connect and Shape” model developed by Whittingham [73], as a positive parenting model following a series of steps in managing child behavior problems. To summarize, first the child is validated emotionally to understand their emotions in relation to the situation. After that, it should be attempted to reorient behavior by facilitating an opportunity to respond adequately, without explicitly solving the problem. The third step consists of paying attention and waiting, at a certain distance, and accepting a new opportunity for molding. If during any of these three steps their behavior comes closer to adequate adaptive behavior, it should be immediately reinforced. 

Homework was to apply this reaction model and identify the barriers found when applying it. Then, they were to keep practicing the exercises and skills they had been training in throughout the protocol, especially considering their actions in the direction of values and full attention exercises. 

#### 2.6.4. Session 4

The purpose of the last session was to provide the parents with some strategies for managing their children’s behavior and emotional problems. We reviewed the actions proposed in “the garden” exercise and valued the importance of continuing to progress in actions for achieving goals even though “the forest is getting dark”. The “Parenting tree” metaphor, which makes an analogy between each part of the tree and aspects of parenting, was introduced. The roots, which give the tree sustenance and sustainability, represent parental values and family dynamics. Emotional competencies are represented as the trunk, which provides a structure and support for the branches of the tree, which represent educational styles and patterns. These points were worked on up to this session in the program. In this last session, concrete educational patterns and styles with flexible positive parenting were worked on to form the tree’s canopy. 

Based on the “Connect and Shape” [73] schema presented in the previous session, the difficulties found during the week of their application were discussed. At this time, all the acceptance, perspective-taking, and mindfulness skills learned in the protocol need to be put into practice to carry out this schema. Options for applying it suitably were given with examples. Different behavioral strategies that could be useful for managing behavior problems were added to it. Specifically, the following behavior modification techniques were practiced: models and modeling, identification of reinforcers, positive and negative reinforcement, control of antecedent stimuli, differential reinforcement, Premack principle, extinction, overcorrection, and timeout. Other educational patterns, such as emotional validation of the children, setting rules and limits, giving orders, and managing information were also practiced.

The session ended by urging the families to keep working on the “Garden” exercise and applying the program skills.

## 3. Results

### Treatment Outcomes and Follow-Up

**Figure 1:**
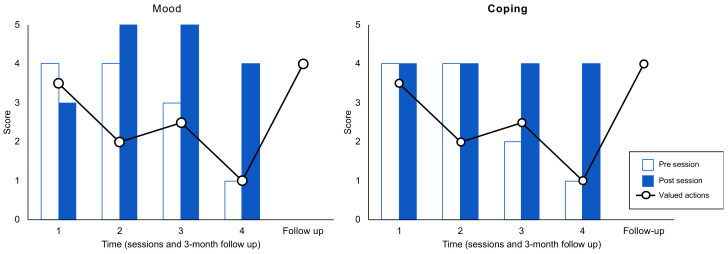
Process outcome of mood, coping ability, and valued actions.

David’s mother’s progress in treatment was positive. Figure 1 shows the mother’s mood and coping progress over the course of the intervention. Mood process outcomes showed an improvement at the end of the sessions from the second one. This increase was especially pronounced in the last session, in which we found the lowest level of mood in the pre-session test. Furthermore, we observed how mood was lower at the beginning of each session (more sadness) and it always improved at the end. Regarding perception of coping, we found maintenance in the two first sessions. Like mood, in sessions three and four we observed the lowest levels of coping at the beginning of each session, which increased at the end to a high level of coping. The largest difference was found in the last session. 

Regarding valued actions (assessed at the start of the sessions), we found a decrease in scores throughout the sessions. It is possible that there is a relationship between these three variables. At follow-up, valued actions score increased to a high level. 

Table 2 shows scores assessed with the instruments described above. We observed an improvement between pre- and post-test score in all variables, except for an aware response style, parenting stressors, and satisfaction with life. Following Jacobson and Truax [52], we found significant clinical changes in total score for emotion regulation skills (DERS total score), emotional acceptance, access to emotion regulation strategies, and baby’s rewards. At the end of the intervention, the mother considered that she should continue improving. She specifically noted, “*when he misbehaves, I would like not to be so negative, because I get very sad*”. She also noted some strategies to regulate herself, such as “*to breathe and think at this moment how much I love him*” and “*I try not to think about the harm he does to me and my family*”.

Regarding satisfaction with the intervention we found a high score (29). On the other hand, protocol exercises and contents of the clinical protocol were rated as very useful (4), satisfactory (4), and it seemed quite easy to carry out (3). 

A follow-up was conducted 3 months after treatment. We found improvements in all variables evaluated except in active response style. These changes are clinically significant [52] in parental psychological flexibility, an open response style, emotion regulation skill (DERS total score), achievement of goals in distress situations, access to emotion regulation strategies, general parenting stress, baby’s rewards, and parental stressors. At follow-up, the mother noted that to regulate herself, she “*thinks of the good times with him*”. 

As for David’s improvements measured with SDQ, we found that the mother perceived a clinically significant change in problems with peers. She also perceived a decrease in emotional symptoms, which was the biggest problem that she scored at the beginning of the intervention. To evaluate David’s emotional well-being, the self-report version of the SDQ was applied. All scores of the SDQ were found in a normal range. Scores were lower for problems with peers (1), emotional symptoms (2), and behavioral problems (2). Prosocial behavior scored high (9). On the other hand, we found clinically significant improvements in experiential avoidance, cognitive fusion, and value-directed actions, which could be interpreted as an increase in psychological flexibility (Table 3).

## 4. Discussion

This article presented a family intervention employing contextual therapies, mainly Acceptance and Commitment Therapy (ACT) [1], to promote psychological flexibility skills and emotion regulation of parents through a case study of a mother and son with behavior problems. The results showed improvement in parental psychological flexibility, emotion regulation, and parental stress. 

The study illustrates the application of various strategies for strengthening acceptance of private events that can appear while managing one’s children’s emotional and behavior problems. Several different exercises were used to promote perspective-taking (or defusion) to make space for this acceptance. One of the main problems that David’s mother had was cognitive fusion with her thoughts. Thoughts such as: “*My son inherited his problem from his grandfather*”, “*All this makes us suffer as a family*”, or “*I am afraid that he’ll end up with a personality problem like my father*”, along with all the emotional burden involved, were lived as if they were real here and now, and furthermore maintained the coherence of a dysfunctional interaction between mother and son, leading to coercive responses in impulsive reactions. Cognitive fusion in parenting can lead to dysfunctional reactions [6]. Most of these reactions can be explained by functional analysis as a pattern of experiential avoidance of these private events which are maintained by short-term negative reinforcement [74,75]. Rumination about parenting is also related to higher stress, which emotional awareness and acceptance skills modulate [76]. The emotional distancing exercises may have promoted psychological flexibility and an open disposition to distressful experiences, reducing avoidance and parental stress. Similarly, Ascanio-Velasco and Ferro García [77] found improvements in the level of stress in a family whose son had behavior problems after applying Parent and Child Interaction Therapy (PCIT) and ACT defusion and valuing elements. 

There was also a context of verbal regulation based on judgements of her son’s good/bad behavior. This led to a diversity of emotional invalidation reactions in David that led to developing similar regulation patterns in him, for example constant rumination about “*everything is shit*” or “*what a shitty life*”. This rumination pattern could be one of the factors involved in David’s aggressive [78] and avoidance behaviors [79]. At the end of the intervention with the mother, a reduction in experiential avoidance, more acceptance, and score within normative ranges on self-perceived behavior problems were achieved. Nevertheless, the mother still thought her son had behavior problems. 

The clinical protocol applied included mindfulness exercises for promoting full attention, both in private events and interactions in family relations [80]. These skills cannot be considered disconnected from those mentioned above. Mindfulness to experience without judging and with a disposition to openness involves exposure that gradually decreases the emotional reactivity [81]. It should also be borne in mind that the reactions to these thoughts are mostly maintained by negative reinforcement, since they continue to palliate in some way the immediate aversive consequences one feels in a certain context, not only behavior (observable), but also by cognitive emotional avoidance processes (unobservable behavior). As in the case illustrated, previous studies have shown that intervention for parents with children with problem behavior employing mindfulness strategies have improved stress levels [82].

Finally, there were significant improvements in the mother’s emotion regulation skills, especially in emotional acceptance, the use of strategies to achieve objectives, and access to emotion regulation strategies in distressful situations. The strategies included are based on emotional validation of the behavior and emotional responses of children and on improvement of emotional awareness using Dialectical Behavioral Therapy (DBT) [83] exercises and strategies for strengthening full awareness and emotional acceptance. In this case, David’s mother referred to several occasions when she had learned to use her “wise mind”, and even made the gesture of taking a step back to remind herself to take perspective. The strategies employed in DBT have already been explored in populations without borderline personality disorder as effective in promoting emotion regulation [84]. Its effectiveness has been explored in some cases of mothers with emotion regulation problems where improvements were found in parenting, in emotion regulation skills, and in stress levels [85,86]. Similar results have been found in parents with children with behavior problems [87]. These results are comparable with those found in this study, in which inclusion of emotion regulation strategies based on acceptance, mindfulness, and defusion achieved similar results. 

In general, all the aspects of flexibility and emotion regulation were framed in a context of values. This makes sense of and motivates the acceptance-based change. The original creative hopelessness processes serve as the basis for generating the motivation to change. From there on, targets and goals are posed from more specific to more abstract terms and are present in the background of the change in motivated behavior in the direction of values [88]. Sometimes, the need for change is motivated by the perception of affection in these vital areas. Specifically, in the case illustrated, the distress associated with “the family”, as a socially constructed value, was given by the problems in family dynamics, which from the mother’s point of view were caused by David. Another aspect worth mentioning found in the process measurements is the relationship observed at the beginning of the sessions between actions in the direction of values during the week, and the mood and perceived ability to cope. Thus, the sessions in which there were less achievement of actions coincided with lower mood and coping.

This study had some limitations. In the first place, the intervention was performed only with the mother, which led us to reflect on the difficulties that would come up in applying these interventions with involvement of both parents, which would be extremely enriching. In this sense, it is a future line that both parents are involved in the intervention. It should be noted that although this study presents the results as a single case, the intervention was performed in a small group, so the results should be considered with this format in mind. Furthermore, the measures employed were mostly self-reported. We tried to palliate this limitation by including several informants and a starting interview. Aside from this, although process measurements were included, this could be improved with ecological momentary assessment [28,89], as well as observational records (both observable and verbal behavior) could be included to assess changes during the therapeutic interaction. Due to design limitations, it was not possible to calculate the effect size; however, to compensate for this limitation, the clinically significant change analysis method was used. Likewise, this study shows an application to a specific disorder, but it would be beneficial to prove its effects on other disorders.

In conclusion, the intervention in this case study showed positive results in improving parental psychological flexibility skills, emotion regulation, and the mother’s stress, both at post-test and follow-up. We also observed improvement in David’s psychological flexibility, with less experiential avoidance and cognitive fusion and more acceptance and willingness to action. These findings suggest the benefits of applying these techniques in family intervention and encourage continuing to explore what each of the factors and components of the protocol contribute.

## 5. Conclusions

### Clinical Implications

This study presents a series of exercises and metaphors which may be applied and adapted to family intervention with third-wave therapies, mainly ACT [1]. The intervention mainly considered aspects related to emotion regulation and flexibility from a perspective of acceptance. Thus, we did not pursue control or change in emotion, but attempted to change how they interacted with these emotions. The use of emotion regulation strategies directed at modifying or changing emotions is common in psychological problems [90], but may provide responses of emotional invalidation, both in the client and in others (in this case, the children). Work on experiential acceptance of emotions and thoughts that arise during parenting or management of behavior problems is therefore of special relevance.

Experiential acceptance makes sense if the intervention is framed within a context of values which decide, within the variability of the behavior, what actions will take one nearer to a life with sense in one’s personal values [88]. It should also be emphasized that the use of metaphors and exercises must not be applied indiscriminately following the protocol word for word, but should be presented in coordination with both observable and verbal clinically relevant behaviors [91]. Similarly, the metaphors and exercises should be directed at strengthening various skills at the same time (acceptance, perspective-taking, full attention), so they should be connected to each other. For example, the exercise on full attention to emotions can also promote acceptance of emotions at the same time. 

Functional analysis of behavior is also present throughout the clinical protocol, both in the son’s behavior as reported by the mother and of the mother herself in her in-session and out-of-session reactions.

## Figures and Tables

**Table 1:** Exercises and objectives of the clinical protocol.

	Contents	O	AW	AC	Objectives
1	The forest road metaphor	x		x	Establish the therapeutic alliance, set out objective of the intervention and introduce value-actions and some mindfulness skills.
	The mind is a lake metaphor		x		
	Mindfulness to breathing		x		
	The garden metaphor			x	
2	The garden metaphor			x	Provide to families for some acceptance-based emotion regulation, mindfulness, and decision-making strategies.
	Body Scan		x		
	Wise mind exercise	x	x		
	The star observatory	x	x		
3	Mindfulness to sounds	x	x		Promote acceptance through mindfulness and cognitive defusion. Take into consideration functional analysis of behavior and associate trained skills with analyzed difficulties. Provide some positive parenting strategies.
	The garden metaphor			x	
	Functional analysis			x	
	The cascade of emotions	x	x		
	Positive parenting			x	
4	The garden metaphor			x	Analyze the difficulties found in the management of misbehavior or emotional dysregulation in children. Provide new tools from behavior modification to manage emotional and behavioral problems.
	The tree of parenting metaphor			x	
	“Connect and Shape” model	x	x	x	
	Behavioral strategies			x	

Note: O—open response style (acceptance + defusion); AW—aware response style (mindfulness + self as context); AC—active response style (actions + values).

**Table 2:** Pre, post, and follow-up scores on the mother.

Variable	Pre	Post	Follow-Up
6-PAQ	34	32 *	29 **
Open	13	12 *	9 **
Aware	13	13	12 *
Active	8	7 *	8
AAQ-II	43	35 *	27 *
DERS	101	79 **	73 **
Attention	7	4 *	4 *
Clarity	7	5 *	6 *
Acceptance	32	24 **	28 *
Goals	17	17	9 **
Strategies	38	29 **	26 **
PSS	40	38 *	30 **
Rewards	16	11 **	8 **
Stressors	24	27	22 **
SWL	14	11	15 *
SDQ	30	30	27 *
Emotional symptoms	7	5 *	4 **
Behavior problems	6	6	6
Hyperactivity	6	6	6
Peers’ problems	3	6 **	4
Prosocial	6	5	5

Note: * clinically significant change improvement; ** clinically significant change recovery.

**Table 3:** Pre and post scores on David’s self-report.

Variable	Pre	Post
WAM	27	16 *
Willingness	5	0 *
Action	22	12 **
AFQ	3.29	1.41 **
Cognitive fusion	3.13	1.25 **
Experiential Avoidance	3.44	1.56 **
SDQ	-	19
Emotional symptoms	-	2
Behavior problems	-	2
Hyperactivity	-	5
Peers’ problems	-	1
Prosocial	-	9

Note: * clinically significant change improvement; ** clinically significant change recovery. SDQ scores were not taken at pre-test.

## Data Availability

The data presented in this study are available on request from the corresponding author.

## Appendix A

**Table A1:** Example of a clinical protocol dialogue with the mother.

M: This week I’m coming in badly. The problem is that it all comes from his grandfather, he’s a video game addict, and if what he wants doesn’t happen, he explodes and is aggressive. T: At what point does this happen? M: All the time, he behaves like this continuously. He challenges me. T: Tell us what happened this week. M: For telling you something… I have them written down, eh? (Looking to her mobile) Since he couldn’t go out on the street when he wanted to, he went to hurt me where he knows it hurts the most. He broke a picture of the amusement park that we lined up for hours. T: And in that moment how did you react? M: Well, yes, I shouted at him… but I couldn’t control myself. T: So, at that time… where were you headed? M: I walked away from my goals, but I couldn’t control it. T: Can you control what you feel? Can we control how those thoughts or emotions move in the whirlpool? M: I try. T: How? M: I try not to think about the damage it does to us, or about my father when all this happens. T: Can we get what we feel out of our minds? M: Yes, if I think of something positive, something else. T: And if I told you right now not to think about a pink elephant, what would you be thinking about? M: The pink elephant. Yeah, but at that moment I can’t see anything clearly. I see everything black. T: Who was in charge? M: Yes, I’ve totally given in to my emotions. T: Then there’s this thought of “he’s doing it to hurt me” “this is all coming from his grandfather” and… M: And then I feel that everything turns around and I answer. T: Who is having that thought at that moment? M: Me. T: So, can you make a choice to do one or the other? M: I don’t know. T: Think for a moment what you could have done in that moment if you used your wise mind. Think about what has stopped you from following your wise mind, from following the path of your forest. M: Yes, I can step back and pay attention to how I feel… T: If you make space for your thoughts and emotions, if you let them be there for a while, we may not necessarily have to respond as if they were there. So, which direction would you go? M: That would bring me closer to my goals.

Note: M: mother, T: Therapist.
